# Dupilumab‐Induced Psoriasis in an Adolescent With Severe Atopic Dermatitis: Successful Management With Abrocitinib

**DOI:** 10.1111/pde.70269

**Published:** 2026-05-18

**Authors:** Grigorios Theodosiou, Åke Svensson

**Affiliations:** ^1^ Department of Clinical Sciences Lund University Lund Sweden; ^2^ Department of Dermatology Skåne University Hospital Malmö Sweden

## Abstract

Psoriasiform eruptions have been described in patients treated with dupilumab. This case report describes an adolescent with severe atopic dermatitis who developed a widespread psoriasiform dermatitis 8 months after starting dupilumab. His therapy was switched to abrocitinib resulting in near‐complete resolution of the psoriatic lesions with good atopic dermatitis control. This case adds to the limited pediatric literature on dupilumab‐induced psoriasis and highlights abrocitinib as an effective therapeutic alternative capable of addressing both conditions concurrently.

To the Editors,

1

A 15‐year‐old Asian male (weight 62 kg) with severe childhood‐onset atopic dermatitis (AD) presented after inadequate response to topical corticosteroids, calcineurin inhibitors, narrowband UVB phototherapy, and methotrexate (Figure 1A). The patient had no personal or family history of psoriasis.

**FIGURE 1:**
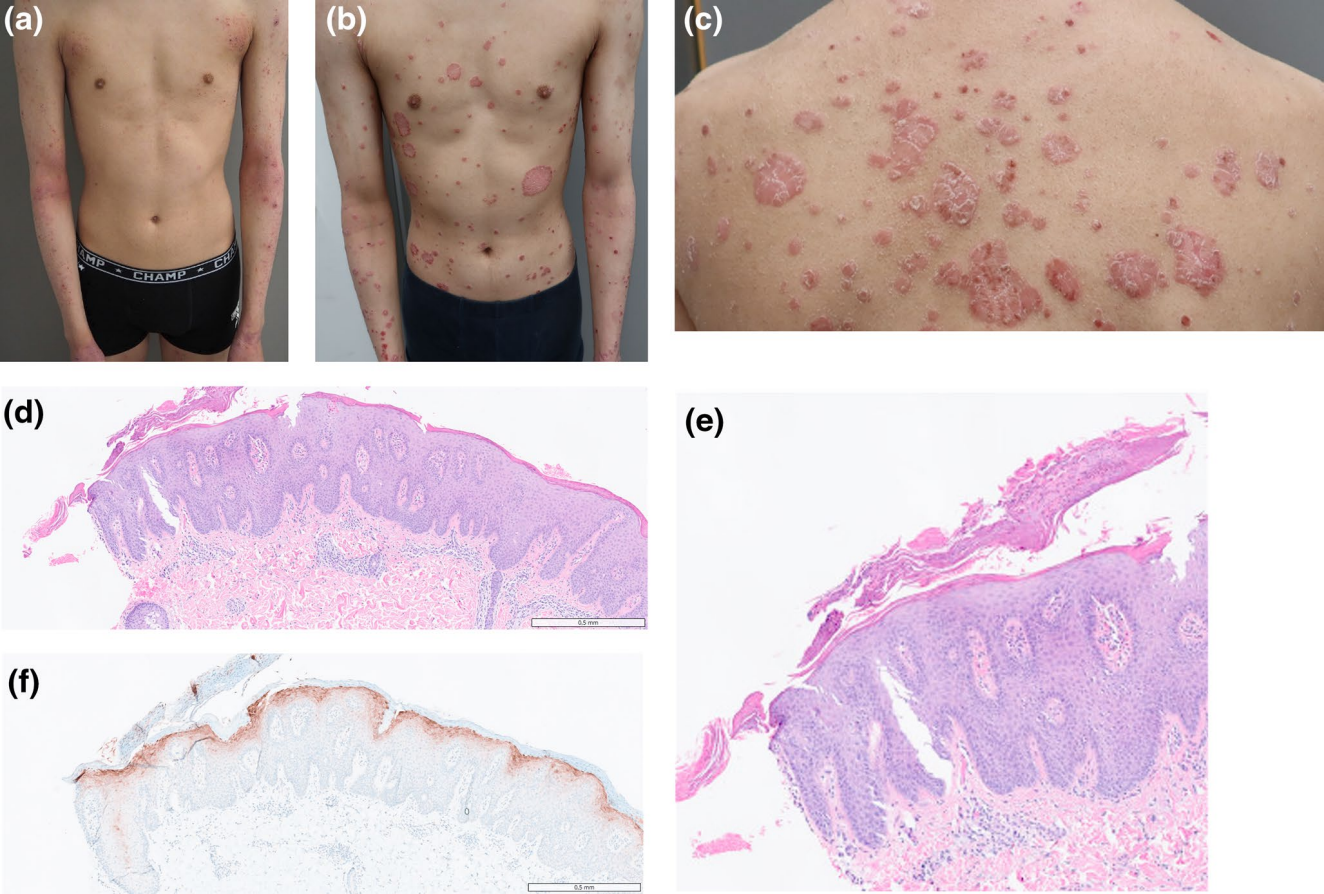
Clinical and histopathological findings in dupilumab‐induced psoriasis. (A) Baseline clinical presentation of severe atopic dermatitis prior to dupilumab initiation, demonstrating widespread erythematous eczematous plaques with excoriations and lichenification. (B–C) Development of well‐demarcated erythematous plaques with overlying silvery scale on the scalp, elbows, and knees after 8 months of dupilumab therapy, clinically consistent with psoriasis. (D–E) Histopathological examination of lesional skin showing psoriasiform epidermal hyperplasia characterized by parakeratosis, loss of the granular layer, regular acanthosis with elongated rete ridges, and neutrophilic collections within the stratum corneum forming Munro microabscesses (H&E, ×100 and ×400). (F) Immunohistochemical staining demonstrating strong expression of IL‐36 within the epidermis.

Dupilumab (600 mg loading dose, then 300 mg every 4 weeks) was initiated, achieving complete remission by week 16 without concomitant systemic therapy.

After 8 months of continuous dupilumab treatment, the patient developed clinically distinct cutaneous lesions. Physical examination revealed well‐demarcated erythematous plaques with silvery scales involving approximately 35% body surface area, affecting the scalp, elbows, torso, and knees (Figure 1B,C). The lesions were notably non‐pruritic and demonstrated a positive Auspitz sign upon removal of scales. Histopathological examination confirmed psoriasiform dermatitis with characteristic features including parakeratosis, absence of the granular layer, regular acanthosis with elongated rete ridges, and neutrophilic collections in the stratum corneum forming Munro microabscesses (Figure 1D,E). Immunostaining showed strong expression of IL‐36 (Figure 1F). Laboratory investigations, including complete blood count, comprehensive metabolic panel, C‐reactive protein, and erythrocyte sedimentation rate, were within normal limits.

Dupilumab‐induced psoriasis was diagnosed based on the temporal relationship and histopathologic findings. Dupilumab was discontinued, and abrocitinib 200 mg once daily was initiated to address both the underlying AD and the treatment‐induced psoriasis simultaneously. Within 2 weeks of starting abrocitinib, psoriatic plaques began flattening with decreased scale formation. By 4 weeks, near‐complete resolution of psoriatic lesions was achieved while AD disease activity remained minimal with no recurrence of eczematous lesions. The patient tolerated abrocitinib well throughout 6 months of follow‐up, with stable safety laboratory parameters including complete blood counts, liver function tests, and lipid profiles monitored at regular intervals.

Dupilumab‐induced psoriasis represents a paradoxical immune‐mediated reaction occurring in approximately 1.7% of treated AD patients, typically manifesting 2–18 months after treatment initiation [1, 2]. The phenomenon is hypothesized to result from inhibition of IL‐4 and IL‐13 signaling, which physiologically suppress Th17 differentiation. Loss of this regulatory effect may permit expansion of Th1/Th17 pathways, promoting psoriasiform inflammation. Bridgewood et al. demonstrated important cross‐regulation between IL‐4/IL‐13 and IL‐23/IL‐17 axes, supporting this mechanistic model [3].

Strong epidermal IL‐36 expression further supports psoriasis‐type immune activation, as IL‐36 cytokines amplify dendritic cell activation and IL‐23/IL‐17 signaling, promoting keratinocyte hyperproliferation and neutrophilic inflammation characteristic of psoriasis. Additionally, AD endotypes vary across populations; Asian patients demonstrate relatively greater Th17 polarization compared with classic Th2‐dominant disease, which may predispose to paradoxical psoriasiform reactions following Th2 blockade [4]. Management is challenging, as discontinuing dupilumab risks AD recurrence while continuation may worsen psoriasis; reported strategies include topical agents, phototherapy, and conventional or biologic systemics, with variable success [5]. Abrocitinib, an oral JAK1‐selective inhibitor, blocks signaling of multiple cytokines including IL‐4, IL‐13, IL‐31, and TSLP through the JAK–STAT pathway, enabling simultaneous modulation of Th2‐mediated atopic and Th1/Th17‐mediated psoriatic inflammation [6, 7]. Similar successful management of dupilumab‐induced psoriasiform eruptions with JAK inhibition has recently been reported using upadacitinib, further supporting this therapeutic approach [8]. Our case demonstrates abrocitinib‘s utility as bridging therapy following dupilumab‐induced psoriasis, achieving rapid response within 2 weeks and sustained improvement over 6 months, with a favorable safety profile.

## Funding

The authors have nothing to report.

## Conflicts of Interest

The authors declare no conflicts of interest.

## Data Availability

Data sharing is not applicable to this article as no datasets were generated or analyzed during the current study.
